# Contextual Cell Death in Adaptive Immunity: Selecting a Winning Response

**DOI:** 10.3389/fimmu.2019.02898

**Published:** 2019-12-17

**Authors:** Alan Herbert

**Affiliations:** Discovery, InsideOutBio, Inc., Charlestown, MA, United States

**Keywords:** intransitive logic, phenotypic plasticity, contextual cell death, trogocytosis, transendocytosis, chaos & non-linearity, CAR (chimeric antigen receptor) T cells, gene therapeutics

## Abstract

Winning the game “Rock, Scissors, Paper” depends on what others do. There is no guarantee that one choice will always win. Does the adaptive immune system use the same intransitive logic to select winners? Here I propose that specialized receptor-ligand pairs, called clicks, initiate contextual cell death to select the best adaptive immune response to a particular challenge. The outcome depends heavily on the phenotypic plasticity of the immune system and upon cell assemblies built from different lineages. These assemblies are self-organizing and use clicks to determine the combination of cells best equipped to defeat a threat. The arrangement is highly adaptive and capable of rapid evolution. Opportunities exist to re-engineer click-based assemblies to produce novel therapeutics.

## Significance

- The immune system bootstraps itself from the Mendelian assortment of receptor-ligand pairs allotted to each individual;
- Certain receptor ligand pairs, here called clicks, direct the contact-dependent killing of one cell-type by another where only one cell survives the interaction;
- The interactions are context dependent and rely on phenotypic plasticity;
- This form of cell death, referred to here as Contextual Cell Death (CCD), differs from Programmed Cell Death and Incidental Cell death;
- CCD selects the best out of many possible immune responses by the targeted fratricide of siblings offering less adaptive responses;
- Responses based on click-based cell assemblies evolve rapidly through the pathogen-driven selection of receptor-ligand pairs that promote survival.

## Outstanding Questions

- Can we reverse engineer clicks to create next generation therapeutics?
- Can we genetically reprogram disease-producing click assemblies?
- Are bispecific therapeutics an effective way to achieve the same goals?
- Can we exploit the phenotypic plasticity of immune cells for similar outcomes?

## Starting From a Single Cell

One of the mysteries of the immune system is that it works at all. The different sets of receptors and ligands inherited from each parent must function together. Adding to this complexity is the rearrangement of immunoglobulin and T-Cell receptor genes to create a repertoire unique to each individual. Further, each possible immune cell varies in how it responds to external stimuli and in the set of genetic programs it expresses. Some cells amplify while others involute. Yet, by working together, immune cells produce a wide range of responses to defeat threats from intra- and extracellular pathogens. Each encounter protects against further attacks without breeching self-tolerance. These outcomes rely on cells that initially know very little about each other, yet together they withstand attacks from a constantly evolving armada of marauders.

**Figure 1:**
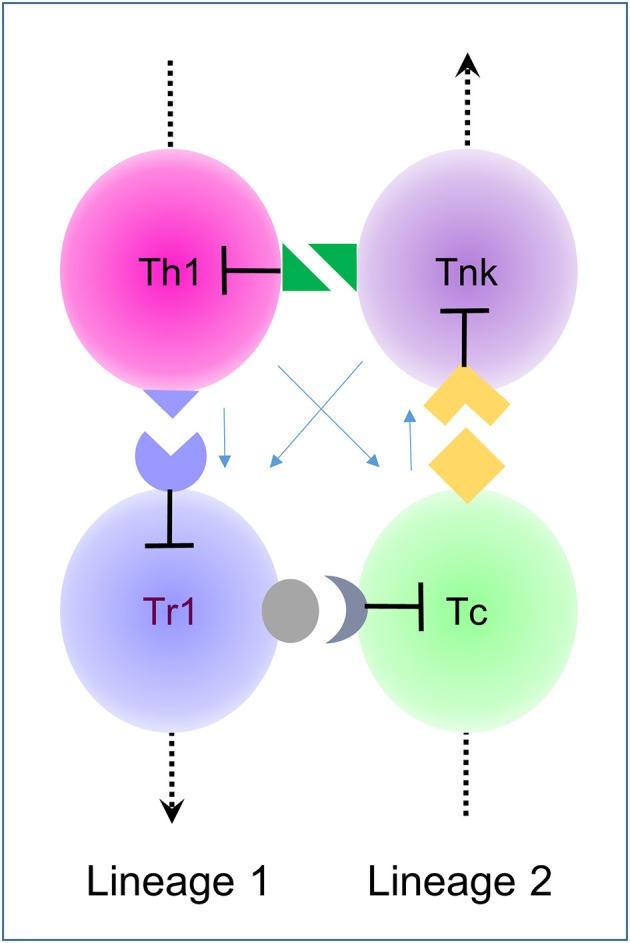
A 4-click based on intransitive logic. Clicks are cell-mediated receptor-ligand interaction between cells in which one partner suppresses the other (lines with flat ends signify killing). Here clicks between type 1 lineage helper cells (Th1 and Tr1) and cytotoxic cells (Tc and Tnk) are illustrated with the arrows on the dotted line pointing from earlier stages of development to later ones (3, 4). This assembly relies on the phenotypic plasticity of both lineages. Depending on context, either a suppressive or cytotoxic immune responses dominates. These assemblies represent directed cycles in which clicks are unique to each cell pairing. Newly generated cells within a lineage regulate an older generation, not its progeny. The light blue arrows indicate bystander parameters that are included in the model and affect the size of each population but do not change the intransitive logic that controls the cycle.

Experimental findings from a wide range of ‘omic technologies demonstrate that immune responses depend upon context and the phenotypic plasticity of the lineages involved. These findings extend the early discoveries of Grossman and Heberman (1) and O'Shea and Paul (2). The more recent data reveals that phenotypic plasticity is more complex than originally imagined, involving, for example, the maturation of T helper cells into T regulatory cells (3), and the expression by activated cytotoxic T-cells of receptors previously thought restricted to the NK lineage (4) (Figure 1). The importance of context in cell fate finds confirmation in single cell sequencing studies, with the probabilities of each outcome changeable by circumstance and influenced by neighbor (5).

## Contextual Cell Death

One approach to modeling immune responses derives from the class of two signal models initially proposed by Brestcher and Cohn (6) and extended by Jain and Pasare (7). The models focus on antigen-specific interactions that provide an activating signal 1, with signal 2 determining whether the triggered cell survives or undergoes programmed cell death. There are three possible outcomes: no response when signal 1 is sub-threshold; stimulation when signal 1 and 2 are both active; either anergy or cell death when signal 1 is present, but signal 2 is absent [reviewed in Baxter and Hodgkin (8)]. Such models build on the clonal selection theory initially proposed by Burnett and describe fate decisions each cell makes for itself [reviewed in Freitas and Rocha (9)]. They do not address the question of how the individual decisions made by each cell combine to select the most appropriate immune response, nor do they address the role of lineage plasticity in outcomes.

The discussion here begins with the many reports of fratricide occurring between immune cells. Such interactions appear to be the rule rather than the exception. Of the estimated, 3 × 10^13^ cells in the human body, 0.3 × 10^12^ are lymphocytes (10). The turnover rate is 0.6% per day (11). Programmed cell death and incidental cell death (caused by external factors, such as severe energy shortage and physical injury) (12) certainly accounts for some of the attrition. Contextual cell death (CCD), where under certain conditions fratricide occurs, is another contributor to cell loss.

Here I propose that CCD is a key regulator of the adaptive immune response. CCD enables the generation of the most pragmatic of all possible solutions to whatever threat presents itself. Selecting the best response involves removing siblings that are less capable of dealing with the threat, those that are best at responding to other types of challenge and those that promote auto-immunity. It is more efficient to remove these siblings than to prescribe exactly how each should behave. Even though this may seem wasteful, there are plenty of replacements produced each day. For the adaptive immune system, selecting from a diverse set of possible responses trumps just using a set of pre-specified endings.

Here I propose a rule set for CCD based on specialized ligand-receptor pairs called “clicks.” Clicks are receptor-ligand pairs formed between two cells that initiate the death of one of the cells. They are directional, acting as triggers that allow one responder population to eliminate another. The definition of clicks is functional and does not refer to a single class of receptor-ligand pair. Examples include antigen-specific receptors that initiate cytotoxic responses and checkpoints inhibitors that produce cell death. The term click is not synonymous with either.

The weapons clicks use for killing cells exist in most, if not all, lineages. For example, perforin- and granzyme-positive granules exist in T-Cells, natural killer (NK) cells, B-cells (13), dendritic cells and polymorphic neutrophils (14). When perforin fails, killing occurs through other cell death pathways such as Fas and Fas ligand (FasL). Some cells execute targets in multiple ways. NK cells deploy perforin/granzyme effectors and death-receptors (15). Cells can trigger death pathways via different clicks. NK use lectin, Fc receptor and other ligands to initiate perforin release. Transendocytosis is another mechanism for inducing cell death. In one example, CTLA-4 strips the co-stimulatory molecules CD80 and CD86 from the membranes of naïve T-Cells, preventing their full activation, condemning them to death by apoptosis (16). In every case, killing is directional, with only one cell dying.

Collectively, these lethal exchanges enable a diverse set of outcomes. Clicks enable the construction of immune circuits from scratch by selective fratricide. The underlying logic is intransitive and depends only on how well a particular cell clicks with its neighbors. Cells that have never met before assemble and decide in a ruthless and efficient manner how to best respond to a threat. The assemblies change adaptively as the threats morph or others materialize. The process depends upon CCD.

## Click Assemblies

A click assembly built with CCD is equivalent mathematically to a directed cycle (Figure 1). In such an arrangement, an arrow drawn from one cell to another results in a circular path that connects neighboring cells. The arrow starts where it ends and it points only one way. There are two rules the click-based assemblies described here: clicks are exclusive to a pair of cells; killing is directional. The logic is conceptually the same as that for the dice game proposed by Effron (17). Like the rock, paper, scissor game, the logic is intransitive (17). The Effron tournament involves a set of four die (corresponding here to four different cell types) where two die are rolled against each other. The die have labels A, B, C and D. Each die shows a different and unique set of numbers. Each number may repeat on more than one facet (these properties correspond here to the restricted and variable expression of click receptors or their ligands). Consequently, rolling one die against another never results in a draw. Yet, it is possible to number the dice so that no one die will win against all others. A die will always exist that defeats another over a series of throws. The relationship is intransitive: If A beats B and B beats C and C beats D, then D will beat A (i.e., a directed cycle). The best way for a contestant to prevail in a Effron match is to pick a die only after the opponent has made their choice. Selecting the right die will guarantee the win. Likewise, a winning immune response depends on the threat. No immune response will defeat all threats, but immune responses specific to a particular threat can arise by using CCD to select the cell assembly most likely to win. Initially, the number of cell types involved may be large and involve many different clicks, but application of the two rules given above will always minimize the number of cells involved. CCD will generate the smallest possible directed cycle and enable selection of the most adaptive immune response (Figure 1).

Figure 1 illustrates an intransitive click assembly based on the different maturational stages of helper and cytotoxic T-Cell lineages. T helper (Th) cells mature into regulatory cells (Tr1) (which are distinct from the Tregs produced from other lineages) (3) while cytotoxic T-cells (Tc) mature into cells that express NK receptors (Tnk) (4). The four cell types connect via receptor-ligand pairs unique to each relationship. The assembly is described here as a 4-click. Young cells connect with older cells from their same lineage while older cells connect to young cells of the other lineage. The interactions are cell-mediated and cause cell death in a directed fashion (the blunt end of the arrow touches the targeted cell). Other factors, such as cytokines and homing receptors affect the size of each population but do not change the underlying logic. The wiring diagrams then only show the relevant clicks, accounting for all other effects by the total number of cells present at each node (Figure 1).

What are the clicks regulating Tc and Th in Figure 1? Tnk likely use NK receptors to kill off activated Th that express a high level of cognate ligands (4). Th in turn kill Tr1 through death-receptor engagement while Tr1 likely kill naïve Tc through interactions involving checkpoint inhibitors, such as CTLA4 dependent transendocytosis (18). The click between Tc and Tnk could involve a genetically encoded receptor-ligand pair. Alternatively, this interaction may require Tc recognition of antigens captured on the Tnk surface the antigen-specific receptor that they share via by the process of trogocytosis (19).

**Figure 2:**
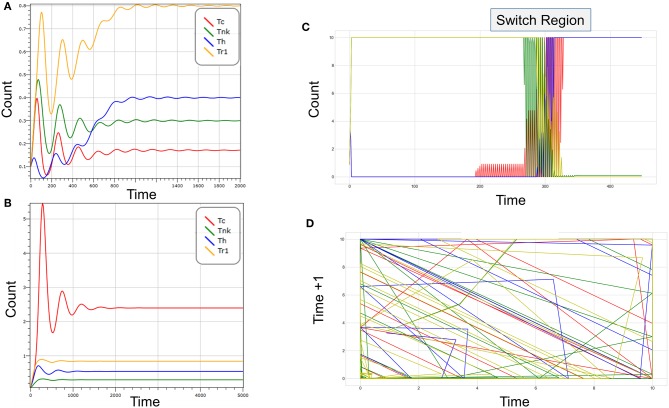
**(A)** A 4-click reaches stable equilibrium over time. In the absence of perturbation, all four populations such as those shown in Figure 1 persist. In this example, P1 corresponds to cytotoxic cells (Tc), P2 to activated cytotoxic cells with NK receptors (Tnk), P3 to T-helper cells (Th), and P4 to T-regulatory cells (Tr1). Tr1 is most frequent in the resting sate (max response ~0.9). The time scale is arbitrary. **(B)** A Tc response develops when the number of Tnk decreases. Fewer Tnk cells allow the Th population to expand. More Th cells leads to increased suppression of Tr1 cells. Loss of Tr1 cells then relieves suppression of Tc. **(C)** The switch between suppressor and cytotoxic responses is chaotic and depends on the level of bystander help. **(D)** A plot of time +1 vs. time reveals that the paths taken during the transition are non-overlapping as is characteristic of a chaotic transition. The color-coding of cells in **(C,D)** is as in **(A,B)**. **(A,B)** were generated using Visual GEC. **(C,D)** were created using a Python script parameterization of the assembly in Figure 1.

How do clicks decide responses? The circuit shown in Figure 1 produces bistable outcomes (Figure 2A), with suppressive or cytotoxic responses dominating, depending on the level of bystander help, the recruitment of different cell-types by tissue-specific factors and the rate of self-renewal of each population. Increasing levels of help drive the expansion of the Tc population along with the Th population (Figure 2B). As the cytotoxic population matures into Tnk, they kill the Th population, not only reducing help but also allowing expansion of Tr1 cells. The Tr1 are then sufficient to kill-off Tc. Computer modeling using either deterministic approaches or cell automata confirm these intuitions (see Methods). Transitions between a stable Tc state and a stable Tr1 state are chaotic (Figure 2C displays the switch region and Figure 2D illustrates the different paths involved in the shift). They depend upon bystander effects that regulate the relative levels of soluble helper and suppressor molecules in the milieu intérieur.

## The Building Blocks: Rules for Click Formation

For a click-based immune system to work, click receptors and ligands need be at a high enough frequency for the relevant cells to find each other and for CCD to occur. There are many ways to satisfy this requirement. The NKG2D receptor is one example where degeneracy of binding to multiple ligands ensures a high probability of engagement against a wide range of threats (20). Degeneracy is also true for CD1-restricted NKT cells, which show limited TCR diversity but combat a wide range of bacterial lipids (21). Other innate immune cells like granzyme B containing MR1-restricted MAIT cells use semi-invariant TCRs to click and respond to a diversity of bacterial vitamin B metabolites (22). Even adaptive immune cells concentrate clonotypes in local neighborhoods to facilitate click formation. They then use trogocytosis to transfer cognate antigens from targeted cells to their cell surface, enabling click-interactions with other cells that have the same antigen specificity (19). Strongly reactive cells load enough antigen on their cell surface to become targets for elimination by the abundant horde of cells with lower affinity receptors.

Most often, click formation stems from the presence of pathogens. However, some clicks depend on activation-dependent expression of one or both genetically encoded binding partners. These clicks often involve self-antigens and are timed either to limit self-reactivity or the response duration. Class II MHC expression on dendritic cells is an example limited to a particular maturational state. Only activated T-Cells display Fas, allowing their execution by FasL-bearing T-cells or their suicide by co-expression of FasL (23). At the extremes, maladaptive assembles exist. In these cases, clicks based on self-antigens augment autoreactivity and chronic inflammatory disease.

## Evidence of Clicks in Action

Intra-vital microscopy reveals the perforin-mediated destruction of antigen-presenting dendritic cells (DCs) with high levels of class II MHC by natural regulatory FoxP3 expressing T-Cells. The DC loss limits immune responses (24). Even with such a high-resolution *in vivo* approach, click-based interactions are difficult to detect as dead cells undergo rapid phagocytosis. In other models, adaptive Tregs kill and prevent the maturation of immature DCs in a non-MHC dependent manner, using perforin combined with a different set of granzymes to prevent the initiation of responses (25). The contrasts between adaptive and natural Tregs in how they use MHC-antigens illustrate the diversity of click assemblies controlling T-cell dependent outcomes (26). In yet another click interaction, NK cells prune DCs with low levels of MHC to enhance T-cell responses (27). NK cells are key players in other click assemblies. A subset of NK cells that requires licensing by neutrophils kills off autoreactive B-Cells via FAS (28). Other NK clicks kill off autoreactive T-Cells and underlie the therapeutic benefit of the interleukin-2 receptor alpha specific antibody daclizumab in multiple sclerosis (29). Collectively, these experimentally determined clicks illustrate the diversity of click assemblies associated with different types of immune response. The findings support the thesis advanced here that there are simple rules governing intransitive cellular interactions that produce CCD and that immune responses are not determined by a preset choreography.

A further experimental test of the click-based assemblies is provided by lymphopenic animal models where immune responses are reconstituted in irradiated animals with a limited number of precursors (30). These experiments model the human Omenn syndrome (OMIM: 603554) where the adaptive immune system repertoire is very limited (30). In both cases, there is lymphopenia and autoimmunity. Traditionally, the outcomes are interpreted on the basis of separate Treg and Tc lineages, each with a different repertoire. Tc and Tregs then arise that respond to a limited and non-overlapping set of antigens. They recognize different cells. Autoimmunity results when no Treg exists to prevent a cell from activating Tc responses. In contrast, with a click-based model, autoimmunity arises stochastically because responses involve only a single click assembly rather than the many possible in a wild-type individual with a high diversity repertoire. Autoimmunity develops when click assemblies stabilize cytotoxic responses rather than suppressing them.

Experimentally, the limiting dilution approach enables the identification of clicks relevant to each possible species-specific click assembly. The expression of stimulatory clicks will correlate, while that of suppressive clicks will anti-correlate. Equivalently, immune response will vary with tumor heterogeneity. Some tumor foci will have active responses while others will manifest immunosuppression. The outcome is more likely when seeding of immune cells to a tumor bed is limited, resulting in the selection of different click assemblies (31).

## Looking From the Past to the Future

The contextual nature and phenotypic plasticity of *in vivo* responses challenge those models based on linear hierarchies that relate immune responses to a prescribed developmental choreography. The focus here is on cell assemblies that form directed cycles (Figure 1) where one population kills off another in a prescribed manner. The assemblies act to maintain self-tolerance, even when click activation is by host antigens. Framing of the predominant response relies on an architecture incorporating phenotypic plasticity and bystander effects. While initially many cells interact, the intransitive logic underlying CCD reduces the assembly to the smallest directed cycle capable of sustaining a response.

The click-based cell assemblies are species-specific and have the capacity to evolve CCD variants rapidly through pathogen-driven selection of different receptor-ligand pairs. Even while transmitting different clicks to subsequent generations, each species exploits the same conserved cellular machinery to direct the context-specific killing of unwanted immune effectors. The clicks selected in each clade boost expansion of the cell populations necessary to deliver an appropriate and protective immune response, ensuring survival of sufficient individuals to proliferate the species.

## Questions

There are still many questions left unanswered. Can we identify biomarkers for the clicks critical to the specific types of CCD, allowing us to reverse-engineer these assemblies? Can we find anti-correlated click pairs that measure transitions from disease states to healthy ones? Do these markers improve our understanding of lineage plasticity? Are they guides for developing better immune therapies?

Can we use clicks to re-engineer the immune system therapeutically? Can we do this through genetic reprogramming of immune cells? We are already moving down this path with chimeric antigen receptor therapy specific for surface antigens on tumor cells (32). How about artificial clicks based on bi-specific T-cell engagers that direct cytotoxic T-Cells to kill a target cell capable of forming pathogenic clicks (33)? Can we reset CCD in autoimmune disease to turn off anti-self-responses or create others to eliminate chronic viral infections or develop some that prevent transplant rejection? To meet these challenges, we only need use the same opportunistic rules embraced by evolution to select winners and eliminate losers. We can begin by exploiting the intransitive logic of contextual cell death to find new remedies.

## Methods

The BETA v2015-0310 version of Visual GEC was downloaded from https://www.microsoft.com/en-us/research/project/genetic-engineering-of-living-cells/#!download. The python script was derived from one describing the belousov-zhabotinsky-reaction (https://scipython.com/blog/simulating-the-belousov-zhabotinsky-reaction/). All scripts are available on request.

## Data Availability Statement

The raw data supporting the conclusions of this article will be made available by the authors, without undue reservation, to any qualified researcher.

## Author's Note

AH is the founder of the company InsideOutBio that is committed to open science and working across disciplines.

## Author Contributions

The author confirms being the sole contributor of this work and has approved it for publication.

### Conflict of Interest

AH is the founder of the company InsideOutBio.

## Glossary

**Bootstrapping:** The process whereby a system loads an initial instruction set, enabling the execution of programs for processing inputs and generating outputs.

**Bystander effects:** These include soluble factors present in the milieu intérieur that act independently of the cell-to-cell contact present in click-based assemblies.

**Programmed Cell Death:** Cell suicide mediated through an intracellular, genetically encoded program (35).

**Contextual Cell Death:** Fratricide occurring under a specific cell of circumstance and mediated through cell-to-cell contact. Only one of the two cells survives the interaction.

**Click**: The subset of receptor-ligand interactions that occur between two cells and initiates the death of one of them. Examples include antigen-specific receptors that initiate cytotoxic responses and those checkpoints inhibitors that produce cell death. The term click is not synonymous with any particular class of receptor. The diversity of clicks indicates that natural selection has found many different strategies to implement the intransitive logic necessary to regulate adaptive immune responses. In any click-based cell assembly, the clicks are unique. Each click is only involved in the interaction between two cell types in the directed cycle. Clicks increase the adaptability of the immune system by increasing the combinatorial diversity of possible assemblies available to form a response.

**Directed Cycle:** A cycle is a continuous circular path that passes through different states but always returns to the initial starting point, regardless of where it starts. A directed cycle only goes one-way, although it can contain some transitions from one state to another that are reversible.

**Incidental Cell Death:** Death caused by external factors, such as severe energy shortage and physical injury (12).

**Intransitive Relationship:** When A > B > C and C > A (for example, when “>” means to defeat as in the game of rock, scissors, paper). A graph of the relationship produces a directed cycle.

**Phenotypic Plasticity**: The changes in cell function and phenotype within a given cell lineage as it matures.

**Transendocytosis:** Stripping of cell surface molecules from one cell and endocytosis by another that requires cell to cell contact (16).

**Transitive Relationship:** When A > B > C, then A > C (for example, when “>” means greater than. For example if A = 3, B = 2, C = 1, the relationship is transitive).

**Trogocytosis:** Stripping of cell surface antigens from an antigen presenting cell by an immune cell (such as a B-, T- or NK- cell),which then presents the antigen on its surface. The process depends upon cell to cell contact (19).
